# Grazing Affects Bacterial and Fungal Diversities and Communities in the Rhizosphere and Endosphere Compartments of *Leymus chinensis* through Regulating Nutrient and Ion Distribution

**DOI:** 10.3390/microorganisms9030476

**Published:** 2021-02-25

**Authors:** Yurong Yang, Siying Chen, Xuefeng Wu, Sajid Iqbal Syed, Irfan Ullah Shah Syed, Beitong Huang, Pingting Guan, Deli Wang

**Affiliations:** Key Laboratory for Vegetation Ecology, Ministry of Education/State Environmental Protection Key Laboratory of Wetland Ecology and Vegetation Restoration, School of Environment, Northeast Normal University, Changchun 130117, China; yangyr422@nenu.edu.cn (Y.Y.); chensy915@nenu.edu.cn (S.C.); wuxf112@nenu.edu.cn (X.W.); Sajidswat85@yahoo.com (S.I.S.); Irfanhashmi347@gmail.com (I.U.S.S.); huangbt228@nenu.edu.cn (B.H.); guanpt994@nenu.edu.cn (P.G.)

**Keywords:** grazing, grassland, rhizosphere, endosphere, bacteria, fungi

## Abstract

Plant-associated endophytic microorganisms are essential to developing successful strategies for sustainable agriculture. Grazing is an effective practice of grassland utilization through regulating multitrophic relationships in natural grasslands. This study was conducted for exploring the effects of grazing on the diversities and communities of bacteria and fungi presented in rhizosphere soils, roots, stems, and leaves of *Leymus chinensis* (*L. chinensis*), based on high-throughput sequencing. Grazing increased bacterial diversity but reduced fungal diversity in plant leaves. Further analysis confirmed that the abundance of Chloroflexi, Gemmatimonadota, Nitrospirota, Sordariales, and Pezizales in plant leaves was increased by grazing. The Bray–Curtis similarities of microbial communities in the endosphere were higher under grazing plots than non-grazing plots. Moreover, the bacterial communities were significantly correlated with ions, while the nutrient and negative ions exhibited strong influence on fungal communities. We concluded that grazing-induced changes of microbial diversities and communities in different compartments of a dominant perennial grass (*L. chinensis*) could be attributed to the nutrient and ion distribution in host plant. The current study highlights the importance of livestock in mediating diversities and communities of endophytic microbes, and will be useful for better understanding the complexity of multitrophic interactions in a grassland ecosystem.

## 1. Introduction

Endophytes refer to microbes (mostly bacteria and fungi) that inhabit internal tissues of plants without causing disease. [1,2]. Most endophytic microbes come from the plant surface and rhizosphere soil, and enter the host plant through stomata, wounds, water holes, or root hairs [3]. They play a vital role in enhancing plant nutrient uptake, improving plant growth, and increasing plant tolerance to harsh environment [4,5,6]. In addition, endophytic microbes may exert a crucial impact on plant diversity and community structure via regulating competition and niche differentiation in resource use among different plants [7,8,9]. Endophytic microbial communities are highly variable in different plant organs, and are strongly influenced by host species and environmental factors, as well as plant physiological and biochemical state [10,11,12]. Therefore, understanding the diversity and community composition of endophytic microbes and the environmental variables that contribute to regulating their community structure has an important significance in highlighting their essential roles for plant growth and ecosystem function.

At present, various endophytic microbes have been isolated from different plant species, and the abundance of endophytes in different tissues of the same plant has been found to be higher than previously expected [10,11,12,13]. The community compositions of endophytic bacteria and fungi are not only influenced by plant species [14], but also greatly vary in different tissues of the same plant [15,16,17]. Environmental factors were considered as main drivers to determine the composition and diversity of endophytic microbial communities, including geographic location, soil type, and climate [18,19]. A previous study showed that the diversity of endophytic fungi in *Ginkgo biloba* changed with the seasons [20]. Franzluebbers [21] suggested that the changes in soil C and N fractions due to endophytic fungi infection were minimal in the relatively short term (60 weeks), while the increase in plant productivity of tall fescue infected by endophytic fungi had been observed in long-term pastures. Similarly, Ren et al. [22] indicated that the community structure of endophytic bacteria was significantly related to soil characteristics, including temperature, pH, carbon and nitrogen content. Revealing the spatial niche difference in the composition of endophytic microbial communities among various tissues and its impact factors may improve our understanding of interactions between plant and endophytic microbes, and promote the effective use of beneficial environmental microbes.

Grazing has been considered as one of the most important management strategies for grassland ecosystems. It is generally accepted that grazing can influence soil microbial community directly, through changing soil properties by animal trampling and dunging, or indirectly, through impacting plant physical and chemical properties by foraging [23]. Despite the importance of endophytic microbes for plant health and ecosystem functioning, it remains unclear whether and how they are impacted by human-caused disturbances, such as livestock grazing. *Leymus chinensis* (Trin.) Tzvel. is a key, dominant C3 grass across eastern areas of the Eurasian Steppe, and is known for its broad distribution and high environment adaptability [24]. As a perennial grass with strong saline–alkaline tolerance, *L. chinensis* has been considered as one of the most promising species for typical grassland rehabilitation in arid and semi-arid regions of northern China [24]. In addition, *L. chinensis* is also an important forage species, preferred by large herbivores because of its high nutritional value and good palatability [25].

Although endophytes are ubiquitous in plants, we know little about their diversity and community structure in different tissues of *L. chinensis*. Furthermore, grazing management is known to be one of the most important strategies for regulating vegetation composition and the nutrient cycle in grassland ecosystem, but there have been few studies about the impacts and mechanisms of long-term grazing management on plant endophytic microbial diversity and community composition. Therefore, checking the effects of grazing on endophyte diversity and community structure is urgently required to reveal the influence of human activities on the relationship between microorganisms and plants. Here, we propose two reasonable assumptions to present possible pathways for grazing to affect endophytic microbial diversity and community composition in plant compartments. Firstly, livestock can injure plants by foraging and trampling behaviors, resulting in the changing of plant metabolism. Endophytic microbes inside plant tissues receive nutrients from host plants to complete their life cycle. The changes in plant metabolism will indeed affect plant nutrient status, thereby exerting influence on the diversity and community composition of endophytic microbes. Secondly, soil microbial community structure is strongly impacted by soil properties, while grazing can influence soil properties by defoliation, treading, and dunging. The changes in soil microbial community composition will in turn regulate endophyte diversity and community composition inside different plant tissues, because endophytic microbes are mainly derived from rhizosphere soil microorganisms. Thus, the main objectives of the current study were to (1) determine endophytic bacterial and fungal diversity and community composition in different plant compartments, (2) explore the effects of grazing on diversity and community structure of endophytes, and (3) unveil the mechanisms how long-term grazing mediates the diversity and community of endophytic microbes in different plant compartments.

## 2. Materials and Methods

### 2.1. Study Area

This study was conducted at the Songnen Grassland Ecosystem Research Station (44°45′ N, 123°45′ E), Northeast Normal University, Changling County, Jilin Province. The study area is located in the south of Songnen Plain, and is part of a meadow steppe with an altitude of 138–167 m. The area has a temperate semiarid continental monsoon climate. with cold and dry winters and warm and rainy summers. The mean annual precipitation is 432.0 mm and the mean annual evaporation is 1368 mm (approximately four times as high as mean annual precipitation). The mean annual temperature is 5.5 °C, and the frost-free period is about 140 days. The most dominant soil type is light chernozem soil with high salinity and alkalization, poor fertility, low organic matter content, and a marked calcic horizon. The vegetation is predominated by *L. chinensis*, and other main companion species are *Calamagrostis epigejos*, *Kalimeris integrifolia*, and *Lespedeza davurica* [26]. Most plant species in the study area green up in early May and senesce in late September.

### 2.2. Sampling

To determine the effects of grazing on plant characteristics, soil properties, diversity, and community composition of endophytic microbes in different plant compartments, the non-grazed (NG) grassland was set up to completely exclude livestock grazing in the meadow steppe from 1999. The NG block, comprising an area of 50 ha, was established as a grazing exclusion district. Inside the NG grassland, the area has been constantly fenced to exclude grazing for more than 20 years. Outside the fence, the grazed (G) grassland (200 ha) suffered different grazing intensities, ranging from two to five cattle (corresponding to roughly to 12–30 sheep) per ha. The domestic sheep could freely eat grass outside the NG grassland during the warm-season.

The experiment was conducted in late July 2019, when regional grasslands were at peak productivity. The NG grassland was surrounded by G grassland. The blocks with similar plant community were selected within 20 ha in NG grassland and G grassland (located in the north of NG grassland), respectively. To avoid the edge influence and the variation in soil type and microclimate, the distance between NG and G grasslands was about 5 km. Both NG and G grasslands were replicated with four blocks (50 × 50 m), and nine plots of 10 × 10 m were randomly established in each block following the line transect method [27]. Subsequently, five 0.5 m × 0.5 m quadrats at the center and four corners of each plot were chosen to investigate the plant height (Supplementary Materials Figure S1). At each quadrat, two soil pits (10 cm × 10 cm) were excavated to a 20 cm depth by a flat spade, and the shoots and roots of *L. chinensis* were separated from other plant species. Rhizosphere soils for each of the pits were collected by gently removing the soil tightly adhered to plant roots, according to the method described by Chaparro et al. [28]. The plant aerial part was clipped close to the soil surface with scissors, and then the whole plant was divided into leaf, stem. and root tissues. The samples within the same block were then mixed thoroughly to one composite sample. All the collected samples were stored in well-sealed zip lock bags and transported in cooled boxes to the laboratory within 24 h. Each sample was divided into two parts: the first was stored at 4 °C prior to the analysis of plant nutrient/ion concentration and soil properties, while the other was stored at −80 °C before DNA extraction.

### 2.3. Plant Physiology and Soil Properties

The tissue samples were dried to a constant weight at 65 °C to obtain dry mass, and then were grinded into powder. The soil samples were ground into a fine powder after air drying and passed through a 100-mesh sieve. Soil pH was determined on a 1:5 soil/deionized water suspension using a portable pH meter (Leici PHBJ-260, Shanghai, China), and soil electronic conductivity (EC) was determined using an electronic conductivity meter (Leici DDS307, Shanghai, China) [29].

The dry tissue samples (0.05 g) were soaked in 10 mL deionized water for 12 h, extracted at 100 °C for 30 min, and centrifuged at 8000 r/min for 10 min, and the content of free inorganic ions was determined with the extract [30,31]. The content of soil potassium ion (K^+^), sodium ion (Na^+^), and calcium ion (Ca^2+^) was tested by flame atomic absorption spectrophotometer (Super-990F); the content of soil sulfate ion (SO_4_^2−^) and chloride ion (Cl^−^) was determined using ion chromatography (DX-300).

The determination of total nitrogen (TN) was tested by the Kjeldahl approach [32]. The nitrate nitrogen (NO_3_^−^–N) and ammonia nitrogen (NH_4_^+^–N) in the soil were extracted with 2 M KCl, and the contents of nitrate nitrogen and ammonia nitrogen in the filtrate were determined by continuous flow automatic analyzer (Alliance-Futura, France) after filtration [33]. The samples were digested in a H_2_SO_4_–H_2_O_2_ mixture at 360 °C [34], after which total phosphorus (TP) was measured colorimetrically using an automatic discontinuous chemical analyzer (AMS Smartchem600, Beijing, China).

### 2.4. DNA Extraction, Amplification, and Sequencing

The tissue samples were washed in running tap water to remove dust and clay from the surface. Next, the surface of each tissue was sterilized by gradually washing in 75% alcohol for 10 s, 0.5% sodium hypochlorite for 8 min, and 75% alcohol for 10 s. Finally, we rinsed the tissue three times with sterilized, distilled water and dried it on sterile paper. The sterilization efficiency was tested by rubbing the sterilized tissue on the surface of the Luria–Bertani (LB) plate and the potato dextrose agar (PDA) plate, to ensure that there were no fungi and bacterial colonies.

Rhizosphere soil and endosphere compartment (plant root, stem, and leaf) samples were grounded to powder with a ceramic pestle and mortar in liquid nitrogen. The total DNA of the rhizosphere soil and endosphere compartment samples were extracted from fresh soil samples using a FastDNA Spin Kit for Soil (MP Biomedicals, Santa Ana, CA, United States). Four biological replicates were manufactured for each tissue and soil sample. The NanoDrop ND-2000 spectrophotometer (Thermo Fisher Scientific, Waltham, MA, United States) was used to measure the concentration and purity of the isolated DNA.

Bacterial 16S rDNA was amplified with primers 338F (5′-ACTCCTACGGGAGGCAGCAG-3′) and 806R (5′-GGACTACHVGGGTWTCTAAT-3′) [35]. Fungal ITS rDNA was amplified with primers ITS1F (5′-CTTGGTCATTTAGAGGAAGTAA-3′) and ITS2R (5′-GCTGCGTTCTTCATCGATGC-3′) [36]. Illumina MiSeq platform (Shanghai Majorbio Science and Technology Co., Ltd., Shanghai, China) was used for PCR amplification and sequencing.

### 2.5. Data Analysis

The raw FASTQ files were demultiplexed and quality-filtered using QIIME2, according to Caporaso et al. [37]. UCHIME was used to identify and remove chimeras from sequences, and then the UPARSE pipeline (version 7.0.1090) was performed to group the filtered sequences into operational taxonomic units (OTUs) with a similarity threshold of 97%, using the SILVA gene database with a confidence threshold of 70%. After filtering out the low-quality reads, trimming the adaptor and primer sequences, and removing the chimeras, we obtained 43,118 and 69,725 highly qualified reads for bacterial endophytes and fungal endophytes in different plant tissues, respectively. The filtered sequences were normalized to 29,872 per sample for endophytic bacteria and 49,434 per sample for endophytic fungi, in order to carry out the downstream analyses among samples at the same sequencing depth. The rarefaction curves of all data sets were calculated to compare the diversity of detected sequences among different compartments based on OTUs defined at 97% similarity. The complexity of endophytic bacterial and fungal diversity among different compartments and the Shannon index were calculated in R software (version 3.6.2) using the vegan package [38]. Non-metric multidimensional scaling (NMDS) was conducted to compare the bacterial and fungal community composition among different compartments, using the “metaMDS” and “adonis” functions in the vegan R package, with a 999 random permutations test (PERMANOVA) based on Bray–Curtis similarity at the OTU level [39]. Redundancy analysis (RDA) was performed using R statistics software (version 3.6.2) within the vegan package to evaluate the relationship between bacterial community structure and environmental parameters [40]. We calculated the Bray–Curtis similarity coefficients for bacterial and fungal communities to compare the differences between compartments using the vegan package [41]. Venn diagrams were constructed to verify the proportion of OTUs that were exclusive and shared among compartments, using the Majorbio I-Sanger Cloud Platform (http://www.majorbio.com) accessed on 2 October 2020. The difference in abundance of bacteria and fungi among different samples was determined at the phylum and order levels using STAMP software, based on a two-sided Welch’s *t*-test and correction by Benjamini–Hochberg FDR [42]. A Mantel test was conducted to assess the correlations between bacterial and fungal community structure and environmental variables, using the ecodist package [43]. The correlation (R matrix) and significance matrices (P matrix) were calculated using Hmsic package in R software (version 3.6.2), and only strong correlations (Spearman’s rank correlation coefficient, *r* > 0.9 (or *r* < −0.9) were selected for further analysis [44]. The constructed correlation matrix was transformed into Gephi software (version 0.9.3) to generate valid co-occurrence networks. Significant difference among the compartments was analyzed using one-way analysis of variance (ANOVA), based on the results from a Levene’s test of homogeneity of variances using SPSS software (version 21). All the sequence data were deposited in the NCBI Sequence Read Archive (SRA) database under accession number SRP302187.

## 3. Results

### 3.1. Community Composition of Endophytic Bacteria and Fungi

**Figure 1:**
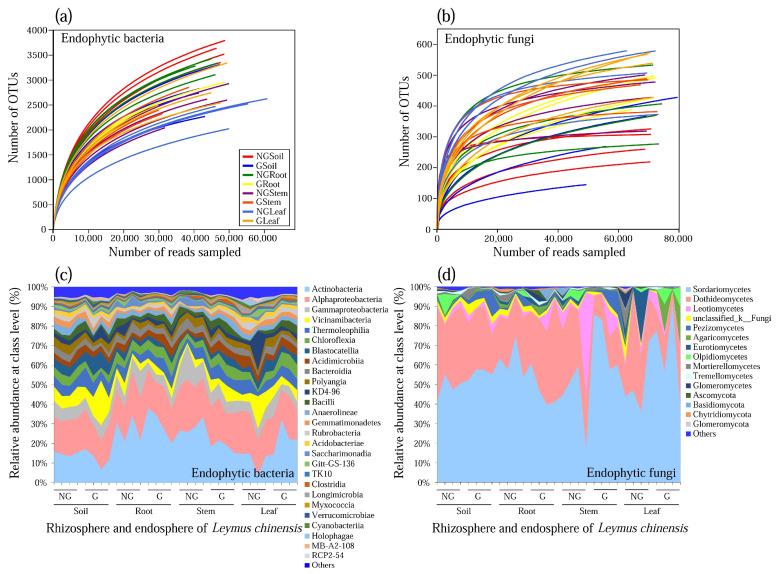
Rarefaction curves of the observed operational taxonomic unit (OTU) number of endophytic bacteria (**a**) and fungi (**b**), and relative abundance of endophytic bacteria (**c**) and fungi (**d**) of *Leymus chinensis* grown in non-grazed and grazed grasslands. NG: non-grazed grassland; G: grazed grassland.

A total number of 7119 operational taxonomic units (OTUs) at 97% similarity were clustered into 40 phyla, 519 families, and 972 genera for endophytic bacteria. In contrast, the total number of OTUs for endophytic fungi were 2195, including 10 phyla, 41 classes, 50 orders, 209 families, and 442 genera. The rarefaction curves tended to reach a saturation plateau, and the coverage was more than 99%, indicating that most bacteria and fungi had been sequenced in all samples (Figure 1a,b). Moreover, the rarefaction curves of fungal endophytes showed that richness of the plant leaf sample (63,500) was generally lower than that of the rhizosphere soil sample (70,703) in grazed grassland.

Forty phyla for endophytic bacteria were observed from different samples, where Actinobacteria, Alphaproteobacteria, Gammaproteobacteria, Vicinamibacteria, Thermoleophilia, and Chloroflexia were the dominant classes, accounting for 62.86% of reads in each sample (Figure 1c). The most abundant OTUs associated with the plant rhizosphere soil library were the sequences related to Actinobacteria and Alphaproteobacteria. At the class level, Sordariomycetes, Dothideomycetes, and Leotiomycetes were the most dominant endophytic fungal communities in all soil and plant tissue samples. The abundance of Actinobacteria in the stem (26.9%) and leaf (28.5%) of the plants grown in non-grazed grassland were almost twice as high as that of plant rhizosphere soil (14.8%) for endophytic bacteria. In contrast, the abundance of Dothideomycetes in the rhizosphere soil (28.4%) and root (38.1%) of the plant grown in grazed grassland were almost twice as high as that of plant leaf (15.0%) for endophytic fungi.

### 3.2. Alpha Diversity of Endophytic Bacterial and Fungal Communities

**Figure 2:**
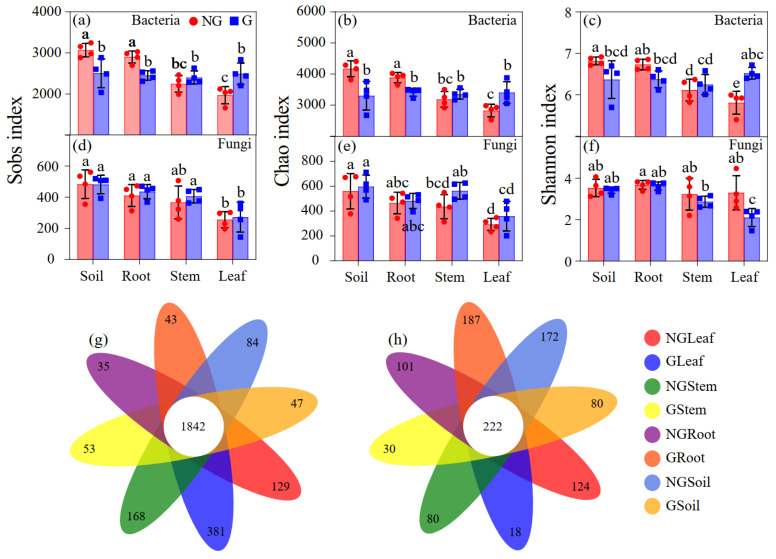
The composition of endophytic bacterial and fungal communities in rhizosphere and endosphere of *Leymus chinensis* grown in non-grazed and grazed grasslands. (**a**–**c**) Plots of Sobs, Chao, and Shannon indices of bacterial community at the OTU level. (**d**–**f**) Plots of Sobs, Chao, and Shannon indices of fungal communities at the OTU level. (**g**,**h**) Venn diagrams of eight groups based on microbial OTU. NG: non-grazed grassland; G: grazed grassland. Different letter indicates significant differences between grasslands (*p* < 0.05).

The observed number of OTUs (Sobs index), as well as the Chao index and Shannon index of endophytic bacterial communities in the leaves of the plants grown in grazed grassland, were significantly higher than those in non-grazed grassland (*p* < 0.05); however, there was no significant difference in Sobs and Chao indices of both endophytic bacterial and fungal communities in the rhizosphere soil and roots of the plants grown in grazed grassland compared with grazed grassland (*p* > 0.05) (Figure 2a–f). Grazing significantly decreased the Shannon index of endophytic fungal communities in plant leaves, but no effect could be found on the Sobs index or the Chao index in any plant tissues (*p* > 0.05).

In total, 7119 bacterial and 2195 fungal OTUs were detected in this study. Additionally, 1842 bacterial OTUs (27.1%) and 222 fungal OTUs (10.1%) were found to be universally present in different compartments of *L. chinensis* (Figure 2g,h). The different tissues of the plant contained 5.0% (leaf), 4.0% (stem), and 1.1% (root) unique bacterial OTUs, and 6.5% (leaf), 5.0% (stem), and 13.1% (root) unique fungal OTUs. The leaf tissue had a greater percentage of unique bacterial OTUs, while the unique fungal OTUs in the stem tissue were significantly lower compared with those in the root tissue.

### 3.3. Difference in Endophytic Bacterial Phylum and Fungal Order among Plant Compartments

**Figure 3:**
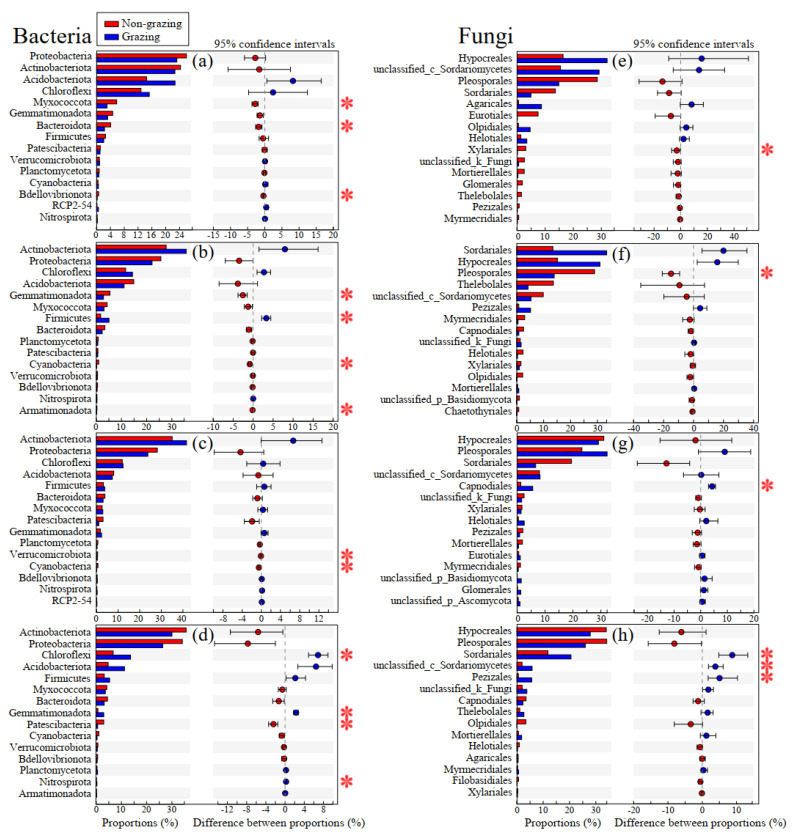
Inter-group difference test of relative abundance of bacteria and fungi in rhizosphere and endosphere compartments of *L. chinensis* at the phylum and order levels, respectively. The relative abundance of bacteria in rhizosphere soil (**a**), root (**b**), stem (**c**), and leaves (**d**) of *L. chinensis* at the phylum level. The relative abundance of fungi in rhizosphere soil (**e**), root (**f**), stem (**g**), and leaves (**h**) of *L. chinensis* at the order level. A two-group comparison was used by a Wilcoxon test with a confidence interval of 95%, with FDR adjustment. NG: non-grazed grassland; G: grazed grassland. * *p* < 0.05.

Pyrosequencing of PCR amplicons and sequences were analyzed based on the SILVA and UNITE databases for bacteria and fungi at the order level, respectively. The results revealed that Actinobacteriota was the most abundant bacterial phylum, accounting for 31.57% of all bacterial sequences in different samples, followed by Proteobacteria (26.15%), Chloroflexi (12.32%), and Acidobacteriota (11.79%). In contrast, Hypocreales (27.06%), Pleosporales (25.23%), and Sordariales (15.47%) were the three most dominant fungal orders when all samples were taken into consideration. The effect of grazing on bacterial and fungal community composition was assessed using a Wilcoxon rank–sum test with FDR adjustment (Figure 3).

### 3.4. Beta Diversity of Endophytic Bacterial and Fungal Communities

**Figure 4:**
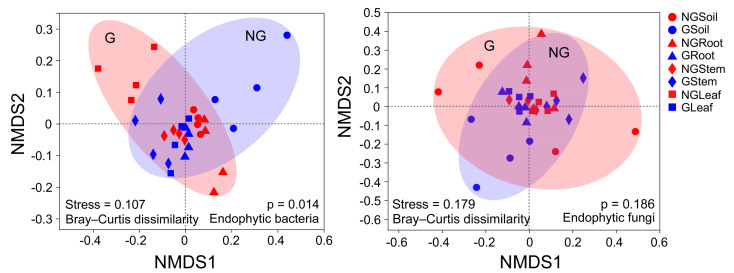
Non-metric multidimensional scaling (NMDS) evaluating differences in the endophytic bacterial community and endophytic fungal community between different tissues of *Leymus chinensis* grown in non-grazed and grazed grasslands. The distance between the samples was calculated based on dissimilarity in OTU composition using the Bray–Curtis dissimilarity index. Points of different shapes represent different samples, and a greater distance between two points represents a higher dissimilarity between them. NG: non-grazed grassland; G: grazed grassland.

Endophytic bacterial community from non-grazed and grazed grasslands were clearly separated into two distinct clusters by NMDS, and the largest distance was found between the GSoil and NGLeaf (stress = 0.107, *p* = 0.014), although the NMDS plots showed many overlaps between samples (Figure 4). However, the ANOSIM analysis revealed no difference in endophytic fungal communities between non-grazed and grazed grasslands (stress = 0.179, *p* = 0.186). Pairwise ANOSIM indicated that the endophytic bacterial communities in different tissues were relatively distinct between non-grazed and grazed grasslands, whereas the community structure of the endophytic fungal community in different tissues was similar between non-grazed and grazed grasslands. Furthermore, livestock grazing increased the Bray–Curtis similarity of bacterial communities in plant stems and leaves, while no significant Bray–Curtis similarity of fungal communities was found among different compartments (Figure S2).

### 3.5. Relationship between the Endophytic Microbial Community and Environmental Variables

**Figure 5:**
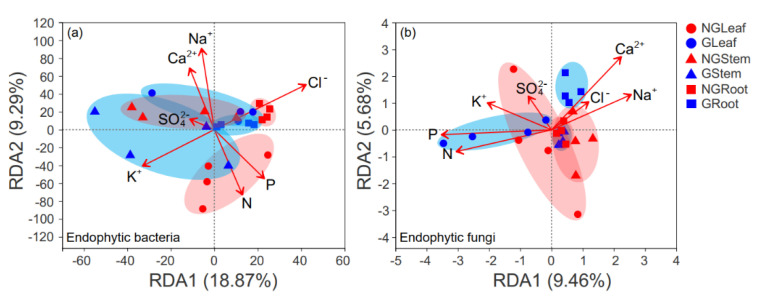
Redundancy analysis (RDA) analysis illustrating the relationship between endophytic microbial communities among different tissues of *Leymus chinensis* and environmental variables: (**a**) endophytic bacterial community, (**b**) endophytic fungal community.

Livestock grazing significantly increased soil pH, EC, NH_4_^+^-N and NO_3_^−^-N concentrations (Figure S3), while reduced soil P concentration (Figure S3). Leaf N, P and K^+^ concentrations were higher in grazed grassland compared with non-grazed grassland, but no significant differences in Ca^2+^, Cl^−^ and SO_4_^2−^ concentrations of plant stems and leaves were found between non-grazed and grazed grasslands (Figure S4). RDA was used to reveal the relationship between the endophytic microbial community at the OTU level among different plant tissues and environmental variables (Figure 5). Plant Na^+^ concentration significantly explained the observed variation in the endophytic bacterial community at OTU level (*p* = 0.039). Plant N and P concentration were detected to be significantly positive to Na^+^ concentration as they were plotted roughly oxygonally (Figure 5a). The first component (RDA1) explained 18.87% of the total variability in the OTU data, implying a distinct gradient of Cl^−^ concentration, whereas the second component (RDA2) explained 9.29% of the variability related to Na^+^ concentration. For endophytic fungal community, the RDA result indicates that environmental factors explained 9.46% and 5.68% of the total variation (Figure 5b). The GLeaf sample was clearly separated from the GRoot sample by the RDA1, and the GStem and NGRoot samples were separated from the Groot sample by the RDA2. GLeaf was more associated with plant N and P concentrations, while GRoot was highly linked to Na^+^, Ca^2+^ and Cl^−^ concentrations (Figure 5b). The mantel test further confirmed that bacterial community was greatly influenced by positive and negative ions, while the fungal community was significantly correlated with the nutrient and negative ions in different plant tissues (Table S1).

### 3.6. Co-Occurrence Network Analysis on Endophytic Bacterial and Fungal Communities

**Figure 6:**
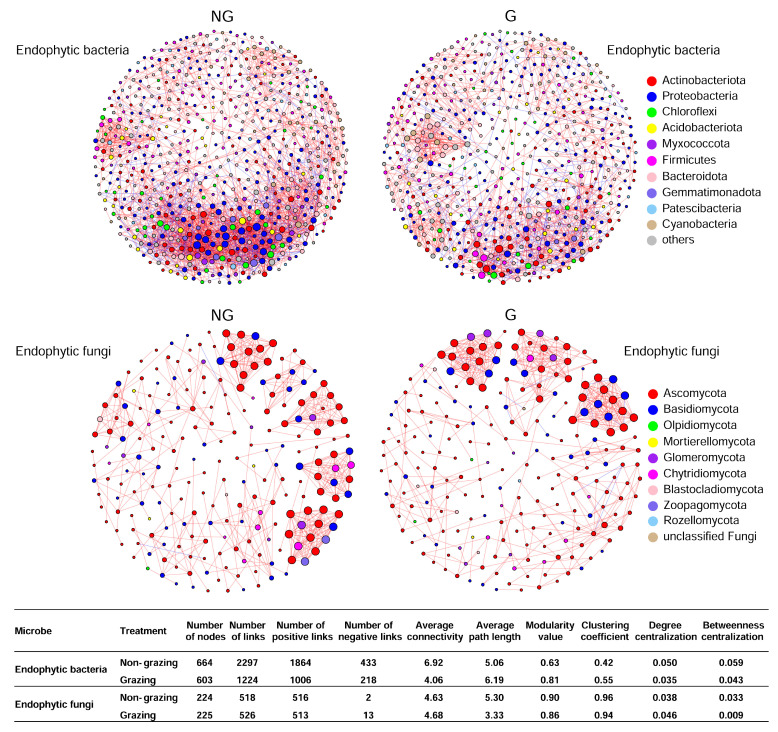
Network analysis showing the interactions in endophytic bacterial and fungal communities of *Leymus chinensis* grown non-grazed and grazed grasslands at the phylum level. Red lines represent significant positive interactions (Spearman’s correlation, *p* < 0.05) between nodes, while blue links represent negative interactions (Spearman’s correlation, *p* < 0.05). NG: non-grazed grassland; G: grazed grassland.

The molecular ecological networks analyses were conducted to reveal the microbial interactions in different tissues of *Leymus chinensis* grown in non-grazed and grazed grasslands (Figure 6). The average connectivity was used to reveal network complexity, and indicated that the endophytic bacterial community in non-grazed grassland (6.92) was more complex than that of grazed grassland (4.06). In contrast, the average connectivity in non-grazed grassland (4.63) was very close to that of grazed grassland (4.68) for the endophytic fungal community. The average path length of endophytic bacterial communities in grazed and non-grazed grasslands were 6.19 and 5.06, respectively. Moreover, the modularity values for endophytic bacterial communities in grazed and non-grazed grasslands were 0.81 and 0.63, respectively, while for endophytic fungal communities the values were 0.86 and 0.90, respectively. These values were all larger than the values calculated by randomized networks, suggesting that this molecular ecological network had typical modular architectures. The numbers of total, positive, and negative links were decreased by grazing for endophytic bacterial community, while the opposite trend was detected for endophytic fungal communities. Interestingly, the numbers of negative links for endophytic fungal communities in grazed grassland was 6.5 times higher than that of non-grazed grassland. However, the number of total and positive links for endophytic fungal communities was similar between non-grazed and grazed grasslands.

## 4. Discussion

### 4.1. Grazing-Induced Changes in Soil Properties and Plant Characteristics

Grazing can directly or indirectly impact soil properties in grassland by regulating the trampling and manure deposition, as well as changing the quantity and quality of litter returned to soil [45,46]. A previous study showed that soil SOC, TN, and TP in the grazed alpine grasslands were significantly lower compared with those in the non-grazed alpine grasslands [47]. In the current study, we found that free grazing significantly decreased soil carbon, TN, and TP in meadow steppes (Figure S2). This result may be attributed to the reduction of litter mass, due to the decrease of vegetation coverage and plant biomass caused by livestock grazing [48]. In contrast, the impacts of grazing on TN and TP from different studies were more variable: positive [49], negative [50], and neutral [51] effects were equally common and varied considerably with grassland ecosystem, grazing intensity, and plant diversity. NH_4_^+^–N and NO_3_^−^–N are the two main inorganic N sources from soil that can be taken up and utilized by most plants. In addition, we found that grazing significantly increased the soil NH_4_^+^–N and NO_3_^−^–N by 27.6% and 54.1%, respectively, which was consistent with a previous study [52]. This could be attributed to the increase of N mineralization caused by the dung and urine deposition of livestock. The free grazing had higher soil pH, EC, and Na^+^, while no differences were found in K^+^ and Ca^2+^ between free grazing and grazing exclusion grasslands. The explanation for these findings should account for the influence of livestock grazing on the increase of soil ion content via urine, feces, and soil compaction [53].

A previous study showed that livestock grazing enhanced N and P concentrations in the leaves, stems, and roots of *L. chinensis* in the Inner Mongolia steppe [54], which was also supported by the results from our study (Figure S4). The increase in N and P concentrations in plant different organs indicate a beneficial effect of grazing on plant growth by improving compensatory growth to reduce biomass loss. McNaughton et al. [55] showed that grazers were able to enhance the return of available forms of N and P to the soil, and thereby increase plant nutrient uptake and accumulation. The present study provides support for this by finding higher soil NH_4_^+^–N and NO_3_^−^–N under free grazing compared with that of grazing exclusion (Figure S3). However, we did not observe any change in plant N/P ratios under free grazing, and this could be attributable to the increase of plant N and P by the same level [56].

### 4.2. Grazing-Induced Changes in Endophytic Microbial Diversity and Community

An increasing number of studies have indicated the crucial effect of microbes in external (rhizosphere) and internal plant compartments (root endosphere, stem endosphere, and leaf endosphere) on plant growth and health [57,58]. The synergistic information about microbial diversity and communities in different compartments of *L. chinensis* and their driving factors are still unclear, although *L. chinensis* is one of the most dominant plant species grown in the eastern region of the Eurasian Steppe, with high ecological and economic values. The lack of this knowledge to some extent hinders our in-depth understanding of the important roles of microorganisms in restoring degraded grassland and maintaining the stability of the ecosystem. Therefore, the characterization of bacterial and fungal diversities and communities in the endosphere compartments and rhizosphere soils of *L. chinensis* is valuable. In the current study, we compared and analyzed the microbial diversity and communities in the rhizosphere soils, roots, stems, and leaves of *L. chinensis* grown in non-grazed and grazed grasslands by Illumina amplicon sequencing. Based on an OTU assessment, the bacterial and fungal diversity in the rhizosphere and endosphere of *L. chinensis* was compared using alpha diversity indices (Sobs, Chao, and Shannon), suggesting higher microbial diversity in the rhizosphere and root compared with stem and leaf tissues in grazed grassland. A rhizosphere is a suitable microenvironment, enriched in nutrients, and contains a huge number and variety of microorganisms [59]. In addition, plant roots can secrete a wide range of chemical compounds that serve important roles in attracting microbes in the rhizosphere [60]. These two reasons might explain the higher microbial diversity in the rhizosphere soil than plant tissues. Rather, the microbes from the soil first colonized plant roots [61], while only some specific species were selected and able to migrate to the aerial parts of plant. With the increase of the distance from the colonized roots, the number of microbes supplied by the xylem gets smaller, resulting in a decrease of richness and diversity of microbial community in this order: root > stem > leaf. These findings were consistent with previous studies in other plant species, such as rice [62], poplar trees [57], and *Medicago truncatula* [58].

Interestingly, there is no difference in the bacterial diversity between rhizosphere and endosphere compartments in grazed grassland. In contrast, the Sobs index and Shannon index of fungal communities in leaf tissue were significantly lower compared with other compartments. We determined the Bray–Curtis similarity of microbial community among different samples to further reveal the role of grazing in shaping microbial community. The grazing tended to increase the similarities of bacterial and fungal community in the endosphere compartment (Figure S2). In other words, the variation in diversity of the microbial community among different compartments become smaller by grazing. Moreover, the Shannon index of the bacterial community was higher in grazed grassland than non-grazed grassland in leaf tissue, while the grazing decreased the Shannon index of fungal communities in the leaf organ. Bacterial colonization could be enhanced by leaf injury caused by livestock feeding [63], resulting in a high number and diversity of airborne bacteria in leaf compartments in grazed grassland. On the other hand, a large number and diversity of bacteria enter the mouth of livestock when they feed on various plant species in the grassland. The bacteria in the mouth are easily migrated to the plants’ wounded leaves through livestock feeding behavior. However, the stronger competition ability of bacteria in plant leaves may greatly inhibit the colonization of fungi.

Our study found that grazing significantly increased the relative abundance of four bacterial phyla (Firmicutes in the root, Chloroflexi, Gemmatimonadota, and Nitrospirota in the leaves) and three fungal orders (Capnodiales in the stem, Sordariales, unclassified_c_Sordariomycetes, and Pezizales in the leaves) (Figure 3). Previous studies have reported that members of Actinobacteria, Proteobacteria, Bacteroidetes, and Firmicutes were commonly detected in various environments and are dominant in the endosphere, with Proteobacteria being the most dominant [64,65]. It has been indicated that Proteobacteria play an important role in fixing atmospheric dinitrogen to ammonia and providing it to the host plant [66,67]. Actinobacteriota have shown various implications, including available nutrient enhancement, plant growth improvement, and phytopathogens inhibition [68,69]. Hypocreales and Sordariales are notable in their ability to promote plant growth by extracting nutrients from multiple sources, and are considered to be important biocontrol fungi for plant herbivores and diseases [70,71]. Our results are in line with Cheng et al. [72], who reported that Firmicutes, Chloroflexi, and Gemmatimonadota were dominant taxonomic groups of soil bacteria in grazed and grazing exclusion grasslands. Because bacterial phyla Firmicutes, Chloroflexi, and Gemmatimonadota have the ability to adapt better to harsh environments [73,74], the changes in the taxonomic compositions were attributed to selective pressure caused by livestock grazing. The increase in the relative abundance of ascomycetes (Pezizales and Sordariales) in grazing plots can be regarded as an adaptation strategy for plants to livestock grazing.

### 4.3. How Grazing Influence Endophytic Microbial Diversity and Community Composition?

**Figure 7:**
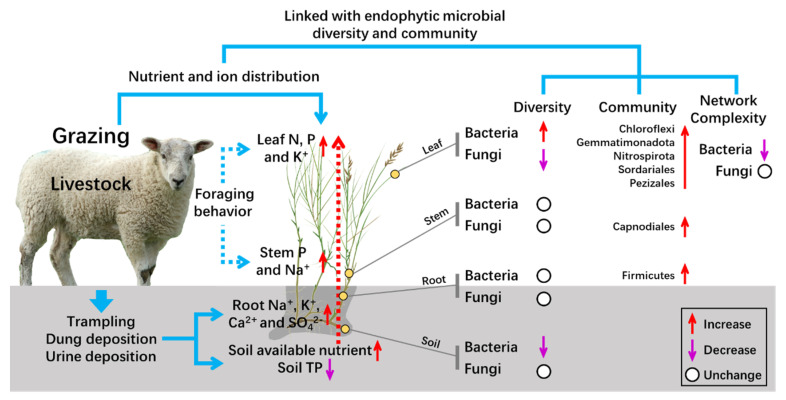
Conceptual framework for understanding the effect of long-term grazing on bacterial and fungal diversity and community in the rhizosphere and endosphere compartments of *L. chinensis*. Livestock grazing increases available soil nutrients, plant nutrients, and ion accumulation through forging behavior, trampling, and dung and urine deposition. The changed soil properties and plant characteristics shape bacterial and fungal communities in different compartments of the plant. Overall, the variation of microbial diversity and community composition in the rhizosphere and endosphere of *L. chinensis* can be attributed to grazing-induced changes in nutrient and ion distribution in the plant–soil system.

Our study suggests that long-term grazing improves soil pH, EC, NH_4_^+^–N, and NO_3_^−^–N concentrations in a meadow steppe in the eastern Eurasian steppe. With the increase of soil available nutrients, plant accumulated more nutrients and K^+^ in the leaves under grazing plots. We further detected that there was no significant difference in Na^+^, Ca^2+^, and SO_4_^2−^ concentrations in plant leaves, although grazing enhanced their concentrations in plant roots. This may be an adaptative strategy for plants related to livestock grazing. A Mantel test was used to reveal the correlation between the microbial (bacterial and fungal) communities and nutrients (N and P), positive ions (Na^+^, K^+^, and Ca^2+^), and negative ions (Cl^−^ and SO_4_^2−^). The study indicates that bacterial communities significantly correlated with positive and negative ions, while the nutrient and negative ions exhibited strong correlations with fungal community (Table S1). As shown by the conceptual framework in Figure 7, this study provided possible scientific values for understanding how long-term grazing impacts bacterial and fungal diversity and community composition in the rhizosphere and endosphere of *L. chinensis* in meadow steppes, based on our own research and previous studies. In a word, grazing affects microbial diversity and community composition in different compartments of *L. chinensis* through regulating nutrient and ion distribution in host plants. However, despite their importance, the rhizosphere and endosphere microbes remain poorly described, and we still lack an understanding of how their diversity and structural compositions are linked their function within different ecological niches. Further studies are required to demonstrate how the grazing-induced changes in microbiota can impact grassland ecosystem function.

## 5. Conclusions

Grazing enhanced the available soil nutrient and ion concentrations, resulting in changes in nutrient and ion distribution among plant tissues. The microbial diversity and community structure were shaped by grazing, and the Mantel test further ensured that the bacterial community was strongly correlated with ions, while the nutrient and negative ions exerted considerable correlations with fungal communities. The interactions among bacterial genera were reduced by livestock grazing, while there was no difference in interactions among fungal genera between NG and G grasslands. Here, we provided a new understanding of the mechanisms involved in the effects of grazing on microbial diversity and community in the rhizosphere and endosphere compartments of a dominant perennial grown in grassland.

## Data Availability

Not applicable.

## Supplementary Materials

The following are available online at https://www.mdpi.com/2076-2607/9/3/476/s1. Figure S1: Location map of region showing non-grazed and grazed grasslands in Changling County, Jilin, China. Figure S2: Community similarity among different rhizosphere and endosphere compartments for (a,b) bacterial communities and (c,d) fungal communities in non-grazed grassland (red circle) and grazed grassland (blue circle). Figure S3: Effects of grazing on soil pH, EC, NH^4+^–N and NO_3_^−^–N concentrations. The difference in soil properties between non-grazed and grazed grasslands was calculated using independent t-test. NG: non-grazed grassland; G: grazed grassland. Figure S4: The nutrient (N and P) and ion (Na^+^, K^+^, Ca^2+^, Cl^−^, and SO_4_^2−^) concentrations of rhizosphere soils, roots, stems, and leaves of Leymus chinensis grown in non-grazed and grazed grasslands. NG: non-grazed grassland; G: grazed grassland. Supplementary Table S1: Mantel tests for the correlation between microbial communities (based on Bray–Curtis distance calculated from an OTU table) and the environmental factors (based on Euclidean distance), using Spearman’s correlation.
