# Supplementary material for: Grazing Affects Bacterial and Fungal Diversities and Communities in the Rhizosphere and Endosphere Compartments of Leymus chinensis through Regulating Nutrient and Ion Distribution
Source: Microorganisms. 2021 Feb 25;9(3):476. doi: 10.3390/microorganisms9030476 (PMC7996188; doi:10.3390/microorganisms9030476)
Supplement: Supplementary file 1 [file microorganisms-09-00476-s001.pdf]

Supplementary Materials

Grazing Affects Bacterial and Fungal Diversities and Communities in the Rhizosphere and Endosphere Compartments of *Leymus chinensis* through Regulating Nutrient and Ion Distribution

Yurong Yang, Siying Chen, Xuefeng Wu, Sajid Iqbal Syed, Irfan Ullah Shah Syed, Beitong Huang, Pingting Guan, Deli Wang*

Key Laboratory for Vegetation Ecology, Ministry of Education/State Environmental Protection Key Laboratory of Wetland Ecology and Vegetation Restoration, School of Environment, Northeast Normal University, Changchun, Jilin 130117, China; yangyr422@nenu.edu.cn (Y.Y.); chensy915@nenu.edu.cn (S.C.); wuxf112@nenu.edu.cn (X.W.); Sajidswat85@yahoo.com (S.S.); Irfanhashmi347@gmail.com (I.S.); huangbt228@nenu.edu.cn (B.H.); guanpt994@nenu.edu.cn (P.G.)

***Correspondence:** wangd@nenu.edu.cn; Tel.: +86-135-0440-3900

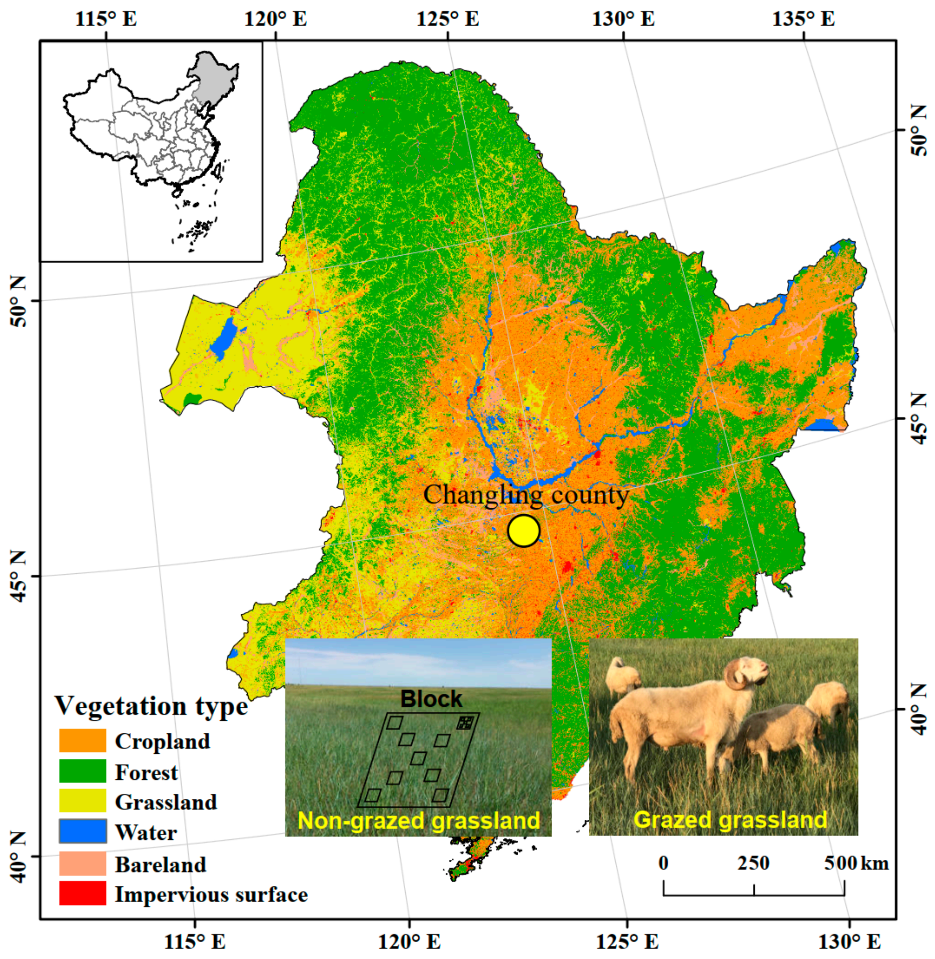

Figure S1. Location map of region showing non-grazed and grazed grasslands in Changling County, Jilin, China.

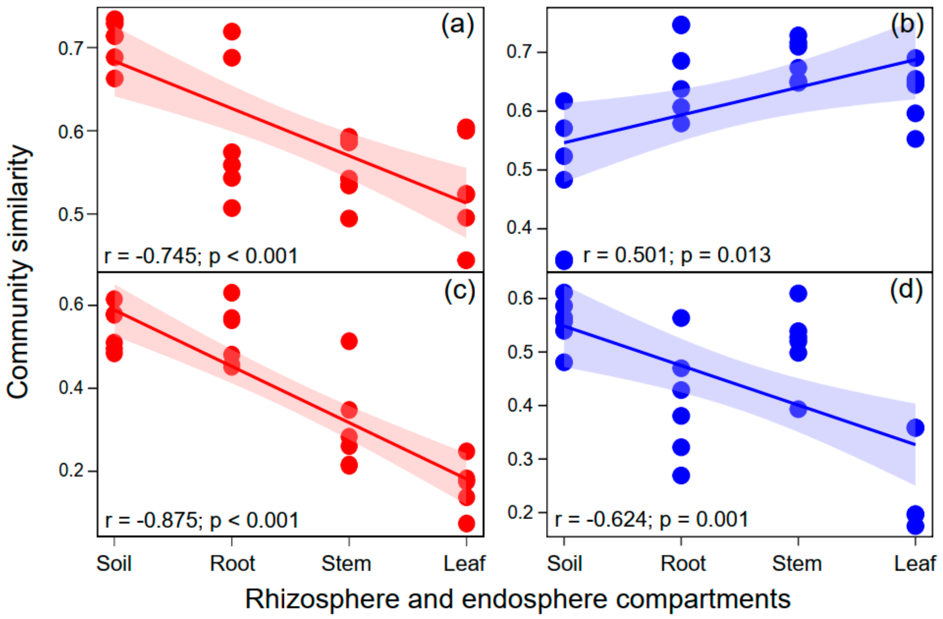

Figure S2. Community similarity among different rhizosphere and endosphere compartments for bacterial communities (a-b) and fungal communities (c-d) in non-grazed grassland (red circle) and grazed grassland (blue circle).

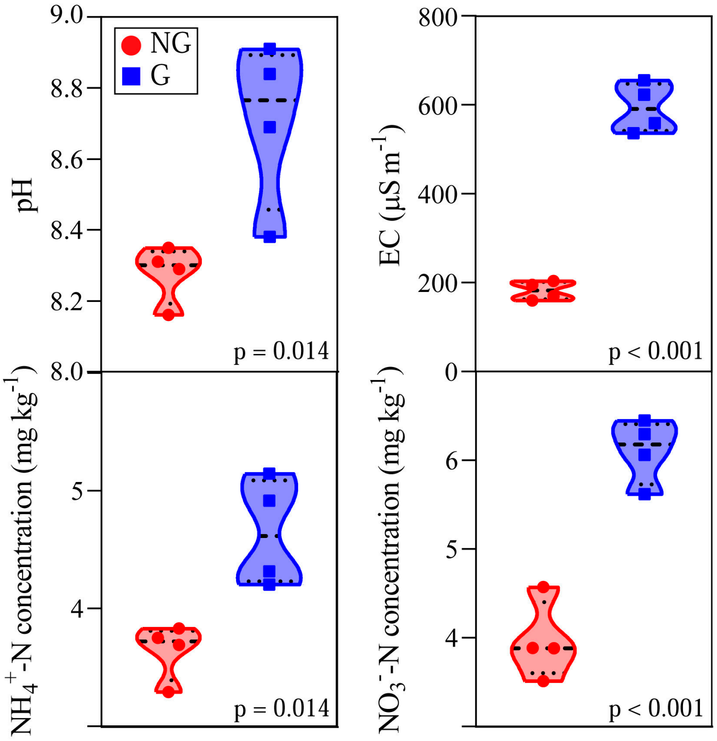

Figure S3. Effects of grazing on soil pH, EC, NH_4^+-N and NO_3^--N concentrations. The difference in soil property between non-grazed and grazed grasslands was calculated using independent t-test. NG: non-grazed grassland; G: grazed grassland.

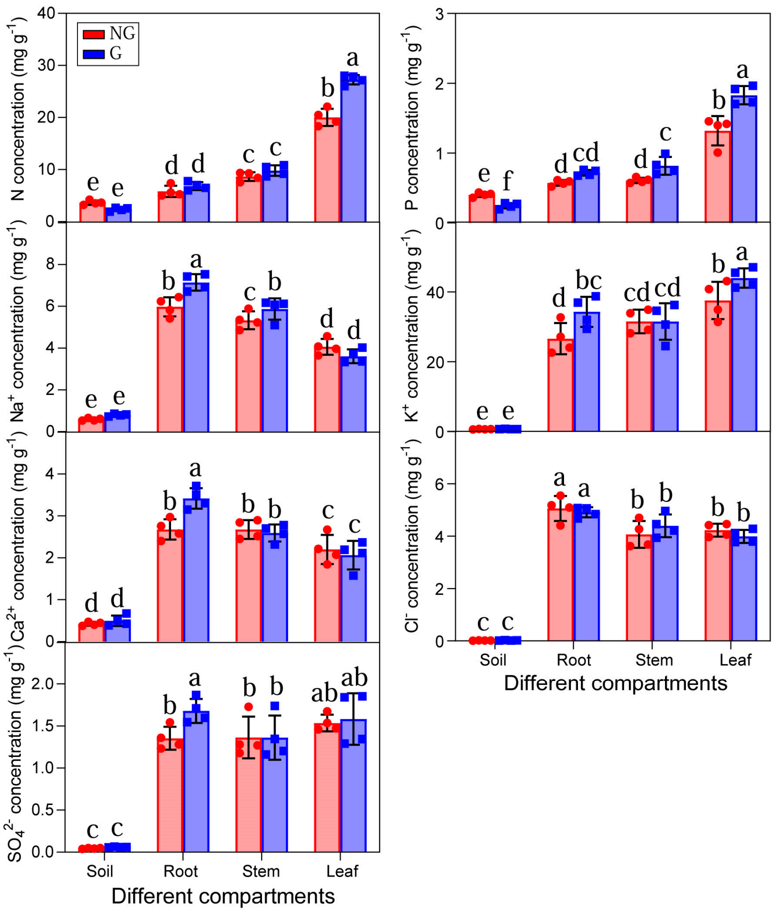

Figure S4. The nutrient (N and P) and ion (Na^{+}, K^{+}, Ca^{2+}, Cl^{-} and SO_4^{2-}) concentrations of rhizosphere soils, roots, stems and leaves of *Leymus chinensis* grown in non-grazed and grazed grasslands. NG: non-grazed grassland; G: grazed grassland.

Table S1. Mantel tests for the correlation between microbial communities (based on Bray-Curtis distance calculated from OTU table) and the environmental factors (based on Euclidean distance) using Spearman's correlation.

Environmental factors	Bacterial community		Fungal community	
	Mantel r	p	Mantel r	p
Nutrient (N and P)	0.104	0.335	0.476	**0.001**
Positive ions (Na^+, K^+ and Ca^{2+})	0.222	**0.009**	-0.041	0.646
Negative ions (Cl^- and SO_4^{2-})	0.178	**0.046**	-0.242	**0.012**
